# *Plebeia catamarcensis* and *Tetragonisca fiebrigi* (Hymenoptera, Apidae) propolis promotes longevity and anti-Alzheimer effects in *Caenorhabditis elegans*

**DOI:** 10.1371/journal.pone.0321487

**Published:** 2025-06-03

**Authors:** Kamila dos Santos Arteman, Paola dos Santos da Rocha, Daniel Ferreira Leite, Alex Santos Oliveira, Igor Victor da Silva, Natalia Guedes Jorge, Alércio da Silva Soutilha, Helder Freitas dos Santos, Debora da Silva Baldivia, José Benedito Perrella Balestieri, Claudia Andrea Lima Cardoso, Kely de Picoli Souza, Edson Lucas dos Santos, Jaqueline Ferreira Campos

**Affiliations:** 1 Research Group on Biotechnology and Bioprospecting Applied to Metabolism (GEBBAM), Federal University of Grande Dourados, Rodovia Dourados-Itahum, Km 12, 79804-970, Dourados, Mato Grosso do Sul, Brazil; 2 Chemistry Course, State University of Mato Grosso do Sul, Rodovia Dourados-Itahum, Km 12, 79804-970, Dourados, Mato Grosso do Sul, Brazil; University of the Pacific, UNITED STATES OF AMERICA

## Abstract

Alzheimer’s disease is a neurodegenerative condition associated with oxidative stress, affecting millions of people worldwide, with expected increases due to the aging population. The development of efficient therapies is still challenging. Propolis, with its antioxidant properties, shows potential in controlling the oxidative stress associated with the disease. The present study aimed to determine the chemical composition of ethanolic extract from propolis produced by *Plebeia catamarcensis* (EEP-Pc) and *Tetragonisca fiebrigi* (EEP-Tf), as well as their *in vitro* and *in vivo* antioxidant effects, lifespan influence and induced paralysis in *Caenorhabditis elegans*, mutant model for Alzheimer’s disease. The chemical profile of EEP-Pc and EEP-Tf was characterized through GC-MS and HPLC, identifying phenolic compounds and terpenes. *In vitro* antioxidant activity was observed using the DPPH• free radicals scavenging method and protection against proteins and DNA oxidation. *In vivo, P. catamarcensis* and *T. fiebrigi* propolis extracts showed no reproductive or locomotor toxicity and promoted resistance to oxidative stress in nematodes exposed to the oxidative agent Juglone. Both propolis extracts increased longevity and reduced the signs of paralysis caused by the accumulation of β-amyloid peptides, a marker for Alzheimer’s disease. Together, these data show that propolis from stingless bees *P. catamarcensis* and *T. fiebrigi* exhibits promising antioxidant activities against oxidative stress, indicating pharmacological potential with the capacity to reduce symptoms of Alzheimer’s disease.

## Introduction

Alzheimer’s disease is a highly progressive neurodegenerative disorder, associated with oxidative stress, and one of the most common causes of dementia (60–70% of cases) [1,2]. Currently, more than 55 million people are diagnosed with dementia worldwide [2]. By 2050, it is estimated that over 152 million people will be diagnosed with Alzheimer’s disease and other neurodegenerative diseases [3], therefore, those conditions are considered a global health problem, with an estimated cost of 2 trillion dollars by 2030 [4].

The disease is characterized by an aggregation of β-amyloid plaques and neurons with neurofibrillary tangles, which promotes neurotoxicity and results in sensory, cognitive, and motor impairments, reducing the quality of life and lifespan of affected individuals [5,6]. β-amyloid plaques emerge from redox imbalance conditions, which also contribute to intensifying neuronal damage [7]. Oxidative stress in Alzheimer’s disease damages neurons’ DNA, proteins, and lipids by interfering with multiple signaling pathways, such as PI3K/Akt, NFκB, RCAN1, CREB/ERK, Nrf2 and PP2A [7,8]. Various drugs and chemical substances derived from natural products, such as resveratrol, genistein, and epigallocatechin gallate, are going through clinical trials due to their role in regulating genes involved in neuroplasticity, neuronal survival, and antioxidant activity [8].

The search for new molecules that improve Alzheimer’s disease symptoms is growing, with a focus on low cost and reduced side effects [8]. Given these issues, the propolis produced by the stingless bees *Plebeia catamarcensis* (HOLMBERG, 1903) and *Tetragonisca fiebrigi* (SCHWARZ, 1938) emerges as potential therapeutic agents for the prevention and/or treatment of this neurodegenerative condition. Propolis is a bee product unique to each species, made by mixing mandibular secretions with botanical exudates [9]. This product has been widely studied for being a complex mixture composed of different classes of metabolites, including phenolic compounds and terpenes, with pharmacological potential [10,11].

To investigate the efficacy of potential therapeutic agents against Alzheimer’s disease, the pre-clinical model *Caenorhabditis elegans* has been widely utilized [12,13]. These animals are known for their high genetic homology with humans in pathways related to oxidative stress and neurodegenerative conditions, making them a promising model for studies regarding the effects and mechanisms of action of natural products [14]. Therefore, the objective of this study was to evaluate the chemical composition of propolis from the stingless bees *P. catamarcensis* and *T. fiebrigi* and investigate their antioxidant effects *in vitro* and *in vivo*, as well as their impact on lifespan and induced paralysis in *C. elegans* mutant for Alzheimer’s disease.

## Materials and methods

### Preparation of propolis ethanolic extract from *Plebeia catamarcensis* and *Tetragonisca fiebrigi*

Propolis samples from stingless bees *P. catamarcensis* and *T. fiebrigi* were collected in the city of Dourados, Mato Grosso do Sul, Brazil (S 22° 13′ 12″ W 54° 49′ 2″). The permission to access genetic heritage was registered under the access code AF5168A in the Sistema Nacional de Gestão do Patrimônio Genético e do Conhecimento tradicional associado (SisGen). The ethanolic extract of propolis from *P. catamarcensis* (EEP-Pc) and *T. fiebrigi* (EEP-Tf) was prepared using 1 g of propolis per 4.5 mL of ethanol 80%. The mixture was heated in a sealed tube in a 70 °C water bath, until total dissolution. The extracts were then filtered using filter paper and stored at -20 °C for later use in experiments [15].

### Chemical analysis

#### Preparation of the samples for GC-MS and HPLC.

EEP-Pc and EEP-Tf (500 µL) were fractioned with hexane and water in a 1:1 (v:v) ratio. The hexane-soluble fraction was analyzed by gas chromatography-mass spectrometry, while the aqueous fraction was analyzed by High-Performance Liquid Chromatography (HPLC).

#### GC-MS.

GC-MS analysis was performed in a gas chromatograph (GC-2010 Plus Shimadzu Kyoto Japan) equipped with a mass spectrometer detector (GC-MS Ultra 2010) utilizing an LM-5 capillary column (15 m length × 0.2 mm i.d. and 0.2 μm thick film) with oven initial temperature set at 150 °C, a heating temperature ranging from 150 °C to 280 °C at 15 °C/min and maintenance at 280 °C for 15 min. Helium gas (99.99%) was used as a carrier gas, with a flow rate of 1 mL/min, a split ratio of 1:10, and a 1 µL injection volume. The injector temperature was set at 280 °C and the quadruple detector temperature was set at 280 °C. The MS scan parameters include a scan interval of 0.3 s, a mass range of 45–600 *m/z,* and an electron-impact ionization voltage of 70 eV. To complete the identifications, the mass spectra were compared in the NIST21 and WILEY29 libraries. In some cases, the compound was identified through comparison with standards. As standards, stigmasterol, β-sitosterol, β-amyrin, lupeol, β-amyrin acetate, and lupeol acetate (Sigma-Aldrich with purity ≥97%) were used in the initial concentration of 1 mg/mL. To determine the compound concentration, external calibration was performed after appropriate dilutions in the range of 0.1–50 µg/mL. The procedure was performed in triplicate.

#### HPLC.

The extracts were analyzed in an HPLC analytical system (LC-6AD, Shimadzu, Japão) coupled with a diode array detector (DAD) monitored at a wavelength between 200 and 400 nm. The HPLC column utilized was C-18 (25 cm × 4.6 mm) with a particle size of 5 μm (Luna, Phenomenex, Torrance, CA, EUA), and to protect the analytical column, a small pre-column (2.5 cm × 3 mm) was used. In each analysis, the injection volume and flow rate were defined as 20 μL and 1.0 mL/min, respectively. The chromatographic analyses were performed at 22 °C. A binary aqueous mobile phase with 6% acetic acid, and 2 mM sodium acetate (eluent A) and acetonitrile (eluent B) were used to perform the elution. The gradients were applied as follows: 5% B (0 min), 15% B (30 min), 50% B (35 min), and 100% B (45 min). An initial concentration of 1 mg/mL of the following standards was prepared: vanillic acid, caffeic acid, ferulic acid, p-coumaric acid, benzoic acid, cinnamic acid, quercetin, luteolin, apigenin, and vanillin (Sigma-Aldrich with purity ≥ 97%). To determine the compound concentration, external calibration was performed after appropriate dilutions in the range of 0.1–50 µg/mL. The procedure was performed in triplicate.

### *In vitro* antioxidant activity

#### DPPH free radical scavenging.

The antioxidant evaluation through 2,2-diphenyl-1-picrylhydrazyl (DPPH•) free radical scavenging was performed according to the method described by Ferreira et al. [16]. For the assay, 200 µL of EEP-Pc or EEP-Tf (2.5–1000 µg/mL) were mixed with 1800 µL of a DPPH• (0.11 mM) solution, diluted in 80% ethanol. The mixture was incubated at room temperature in the dark for 30 minutes. Subsequently, the absorbance was measured at 517 nm in a spectrophotometer (T70 UV/ Vis, PG Instruments Limited, Leicestershire, UK). As reference antioxidants, ascorbic acid and butylated hydroxytoluene (BHT) (2.5–1000 µg/mL) were utilized. The DPPH• free radical scavenging percentage was determined from the control (DPPH• 0.11 mM solution) according to the following equation:


$$\text{DPPH}•\text{free radical scavenging activity }\left(\text{\%}\right)=1-\frac{Abssample}{Abscontrol}\times 100$$ (1)

Three independent assays in triplicate were performed, and the values of the concentration necessary to inhibit 50% of the free radical (IC_50_) were calculated.

#### Protein oxidation.

The effect of the extracts on protein oxidation was investigated using bovine serum albumin (BSA) as a standard, according to the method described by Monteiro-Alfredo et al. [17], with modifications. For the assay, 1.5 μg/mL of BSA was pre-incubated with 15 μL of EEP-Pc or EEP-Tf (50–1000 µg/mL) for 30 min. Subsequently, 15 μL of oxidizing agent AAPH (75 mM) was added and the samples were incubated at 37 °C for 2 h. After incubation, sample buffer was added to the samples, which were heated at 95 °C for 3 min and applied onto a 12% SDS-PAGE polyacrylamide gel. Gels were subjected to electrophoresis in a Mini-PROTEAN Tetra Cell (Bio-Rad Laboratories, CA, USA) system at 200 V for 60 min. Gels were then scanned utilizing the Gel Doc EZ Imager (Bio-Rad Laboratories) photodocumentator. The band’s internal volume was determined using the Image Lab software. The decrease in band volume was considered indicative of protein oxidative damage. Three independent experiments were performed in triplicate and the normalized results were expressed as BSA integrity (%).

#### DNA oxidation.

The assay to investigate the antioxidant activity through inhibition of DNA oxidation was performed as described by Monteiro-Alfredo et al. [17], with slight modifications. For this, K562 cell line genomic DNA (20 ng/μL), diluted in phosphate saline solution (PBS), was mixed with EEP-Pc or EEP-Tf (50–1000 µg/mL) and oxidizing agent hydrogen peroxide (H_2_O_2_) 30%. The samples were incubated in a UVT-312–302 nm transiluminator, for 5 min at room temperature. Subsequently, they were applied to a 2% agarose gel with ethidium bromide (10 mg/mL) and electrophoresis was performed. Gels were then scanned using the Gel Doc EZ Imager (Bio-Rad Laboratories) photodocumentator and analyzed with Image Lab software. Three independent experiments were performed and the mean of 3 gels was used to determine the DNA integrity (%).

### *In vivo* assays

#### Culture, strains, maintenance, and synchronization of *Caenorhabditis elegans.*

For the *in vivo* experiments, *C. elegans* nematodes from the N2 wild strain and the Alzheimer’s disease model strain CL2006 (dvIs2 [pCL12(unc-54/human Abeta peptide 1–42 minigene)+rol-6(su1006)]), were used. The strains were obtained from the *Caenorhabditis* Genetics Center (University of Minnesota, Minneapolis, MN, USA). The N2 nematodes were maintained in incubators at 20 ºC and the CL 2006 strain was kept at 15 ºC. They were cultivated in Petri dishes containing Nematode Growth Medium (NGM) agar with *Escherichia coli* (OP50), which was inactivated using 10 mM kanamycin [18]. For the experiments, the nematodes were synchronized with 2% sodium hypochlorite and 5 M sodium hydroxide. Eggs resistant to the alkaline lysis were collected and transferred to new Petri dishes containing NGM with *E. coli* and were incubated for 48 hours until they reached the L4 stage [18].

#### Sub-chronic toxicity.

To assess the toxic effect of EEP-Pc and EEP-Tf on N2 nematodes, the sub-chronic toxicity assay was performed according to the method described by Leite et al. [19]. For the experiment, an average of 10 nematodes synchronized at the L4 stage, were transferred to 96-well microplates containing 100 µL of M9 buffer and 100 µL of EEP-Pc or EEP-Tf (10–1500 µg/mL), and they were incubated at 20 °C for 24 and 48 h. As a negative control, 200 µL M9 buffer was used. Worm viability was assessed after the incubation period under a stereomicroscope (Motic SMZ-140 & W10X/23), by touch sensitivity utilizing a platinum wire. Three independent assays were performed in triplicate.

#### Reproductive toxicity.

To assess the EEP-Pc and EEP-Tf effects on the reproductive capacity of N2 worms, the method described by Araújo et al. [20] was performed with slight modifications. For the assay, 10 N2 nematodes synchronized at the L4 stage were transferred daily to new Petri dishes containing NGM with 100 µL of EEP-Pc or EEP-Tf (500 and 1000 µg/mL), diluted in *E. coli* OP50. The dishes were incubated at 20 °C. As a negative control, 100 µL of *E. coli* OP50 was used. The number of viable progeny was quantified during a five-day reproductive period, under a stereomicroscope (Motic SMZ-140 & W10X/23), after the worms reached the L3 or L4 larval stage. Two independent experiments were performed in duplicate.

#### Locomotor toxicity.

To evaluate the toxic effect of EEP-PC and EEP-Tf on the locomotor capacity of N2 worms, the method described by Araújo et al. [20] was performed, with assessments occurring in two different stages of the worm life-cycle, on the second and seventh day after they reached the L4 stage, corresponding to the adult and aging phases, respectively. For this assay, an average of 10 N2 nematodes synchronized at the L4 stage were transferred daily to fresh Petri dishes containing NGM agar with 100 µL of EEP-Pc or EEP-Tf (500 e 1000 µg/mL) diluted in *E. coli* OP50. The dishes were incubated at 20 °C. As a negative control, 100 µL of *E. coli* OP50 was used. On the second and seventh days after the worms reached the L4 stage, they were transferred to new plates containing only NGM medium, followed by 1 minute of acclimation with subsequent evaluation under a stereomicroscope (Motic SMZ-140 & W10X/23) to count the number of full bends during 30 seconds of locomotion. Two independent experiments were performed in duplicate.

#### Oxidative stress resistance.

To assess the antioxidant effects of EEP-Pc and EEP-Tf on *C. elegans*, the method described by Leite et al. [19] was followed, with modifications. For the experiment, an average of 10 N2 worms synchronized at the L4 stage were transferred to 96-well microplates containing 100 µL of M9 buffer and 100 µL of EEP-Pc or EEP-Tf (50–1000 µg/mL), with subsequent pre-incubation at 20 °C for 1 h. Following pre-incubation, 50 µL of oxidizing agent 5-Hydroxy-1,4-naphthoquinone (Juglone 40 µM) was added. The nematodes were incubated at 20 °C for 24 and 48 h. As a negative control, 250 µL of M9 buffer was used, while 200 µL of M9 buffer with 50 µL Juglone 40 µM used as an oxidant control. Worm viability was assessed after each incubation period under a stereomicroscope (Motic SMZ-140 & W10X/23) by gently touching them with a platinum wire. Three independent assays were performed in triplicate.

#### Lifespan.

To evaluate the effect of EEP-Pc and EEP-Tf on the lifespan of N2 *C. elegans*, the method described by Leite et al. [19] was followed with minor modifications. For this, 10 N2 nematodes synchronized at the L4 stage were transferred to Petri dishes containing NGM medium with 100 µL of EEP-Pc or EEP-Tf (50–1000 µg/mL), diluted in *E. coli* OP50. For 6 days, after the L4 stage, corresponding to the reproductive period, the worms were transferred to fresh plates daily. Afterward, the nematodes were transferred to new agar plates every 2 days. The plates were kept in incubators at 20 °C. As a negative control, 100 µL of *E. coli* OP50 was utilized. The viability was assessed daily under a stereomicroscope (Motic SMZ-140 & W10X/23) by gently touching them with a platinum wire until all nematodes had died. Two independent assays were conducted in triplicate.

#### Induced paralysis in *C. elegans* mutant for Alzheimer’s disease.

To evaluate the effect of EEP-Pc and EEP-Tf on *C. elegans* mutants that exhibit a paralysis phenotype—an Alzheimer’s disease model—the method described by Sangha et al. [21] was conducted with slight modifications. For the assay, an average of 10 CL2006 worms synchronized at the L4 stage were transferred to Petri dishes containing NGM medium with *E. coli* OP50 with or without the extracts and kept at 25 °C for 22 hours. At this temperature, CL2006 adult nematodes express the paralysis phenotype due to the formation of β-amyloid peptides in the muscle cells of the body wall. After the incubation period, nematodes were evaluated under a stereomicroscope (Motic SMZ-140 & W10X/23) every 2 hours, for 10 hours. The nematodes were considered paralyzed when they did not move their bodies when touched with a platinum wire and when they formed a bacterial halo. Three independent assays were performed in triplicate.

### Statistical analysis

Results are expressed as mean ± standard error of the mean (SEM). To compare the experimental groups, analysis of variance (ANOVA) followed by Dunnett’s test was utilized. The *C. elegans* survival curve was analyzed using the log-rank (Mantel-Cox) test. All statistical analyses were performed using the GraphPad Prism 8.4.3 (San Diego, CA, USA) software. The results were considered significant when *P* < 0.05.

## Results

### Chemical composition

The chemical composition of the ethanolic extracts of propolis from *P. catamarcensis* (EEP-Pc) and *T. fiebrigi* (EEP-Tf), was analyzed, quantifying 14 compounds, belonging to phenolic compounds and terpenes classes. In EEP-Pc, 9 compounds were identified, with phenolic compounds as the major group. Among them, vanillic acid was the most abundant (Table 1). In the EEP-Tf, 12 chemical compounds were identified, with terpenes, such as lupeol, β-amyrin acetate, β-amyrin, and lupeol acetate standing out (Table 1 and S1 Fig).

**Table 1:** Chemical composition of the ethanolic extract of propolis from *P. catarmacensis* (EEP-Pc) and *T. fiebrigi* (EEP-Tf) by GC-MS and HPLC.

Compound	Retention time (min)	Molecular mass	EEP-Pc (µg/mL)	EEP-Tf (µg/mL)
***Phenolic compounds***				
Caffeic acid	8.64	180	–	19.1 ± 0.6
p-Coumaric acid	13.48	164	13.5 ± 0.2	23.9 ± 0.7
Ferulic acid	17.28	194	–	14.7 ± 0.2
Quercetin	35.33	302	5.1 ± 0.1	10.6 ± 0.4
Luteolin	36.68	286	–	12.1 ± 0.3
Apigenin	42.62	270	–	8.8 ± 0.1
Vanillic acid	7.95	168	48.7 ± 1.3	–
***Terpenes***				
Stigmasterol	17.02	412	18.4± 0.2	25.1 ± 0.1
β-Sitosterol	17.72	414	13.1± 0.1	21.4 ± 0.1
β-Amyrin	17.89	426	24.1 ± 0.5	41.3 ± 0.4
Lupeol	18.89	426	20.1 ± 0.6	42.4 ± 0.4
β-Amyrin acetate	19.67	468	18.4 ± 0.7	42.1 ± 0.3
Lupeol acetate	20.74	468	22.9 ± 0.5	41.1 ± 0.3

Values are expressed as the mean ± SEM. Analyses were performed in triplicate.

### *In vitro* antioxidant activity

#### DPPH^•^ free radical scavenging activity.

Given the presence of potential antioxidant substances in both EEP-Pc and EEP-Tf, an *in vitro* evaluation of free radical scavenging activity was performed. Both extracts showed antioxidant activity; however, EEP-Pc presented a lower inhibitory concentration for 50% (IC_50_) of the free radicals compared to EEP-Tf (Table 2).

**Table 2:** *In vitro* DPPH^•^ free radical scavenging activity of ethanolic extract of propolis from *P. catamarcensis* (EEP-Pc) and *T. fiebrigi* (EEP-Tf).

	DPPH^•^		
Samples	IC50 (µg/mL)	Maximum inhibition	
		%	µg/mL
AA	6.51 ± 0.67	87.52 ± 1.36	10
BHT	13.73 ± 3.05	88.34 ± 2.14	50
EEP-Pc	293.56 ± 8.66	68.59 ± 4.32	1000
EEP-Tf	542.7 ± 30.27	62.79 ± 8.35	2000

IC_50:_ Concentration capable of inhibiting 50% of the DPPH^•^ free radical; AA: Ascorbic acid; BHT: butylated hydroxytoluene. The values are expressed as the mean ± SEM.

#### Protection against protein oxidation.

**Fig 1:**
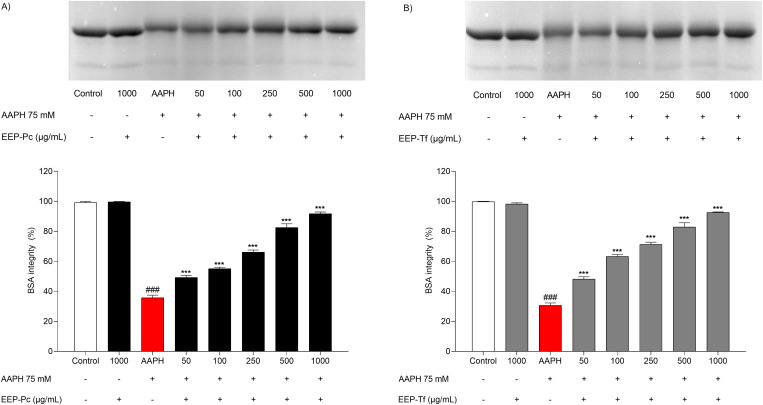
Effect of ethanolic extract of propolis on the integrity of bovine serum albumin (BSA) incubated with oxidizing agent AAPH: (A) *P. catamarcensis* (EEP-Pc) and (B) *T. fiebrigi* (EEP-Tf). The results were generated from the values of 4 independent gels. The results are expressed as mean ± SEM. ^###^*P* < 0.001 versus Control; ***P < 0.001 versus AAPH control.

The propolis extracts mitigated the oxidation of bovine serum albumin (BSA) in a concentration-dependent manner (Fig 1A-B). The AAPH control treatment significantly reduced BSA integrity to about 35% when compared to the negative control. Both extracts preserved BSA integrity, achieving levels close to those of the negative control at the highest concentrations evaluated. EEP-Pc was able to maintain BSA integrity at 82.55 ± 2.48% and 91.76 ± 1.16%, at concentrations of 500 and 1000 µg/mL, respectively (Fig 1A). EEP-Tf maintained BSA integrity at 82.85 ± 2.84% at 500 µg/mL and 92.57 ± 0.39% at 1000 µg/mL (Fig 1B).

#### Protection against DNA oxidation.

**Fig 2:**
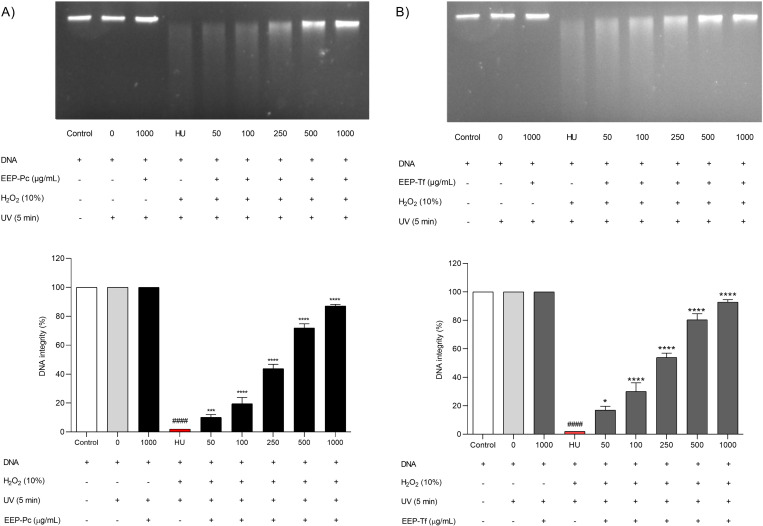
Effect of ethanolic extract of propolis on genomic DNA integrity under oxidative stress induced by H₂O₂ and UV radiation: (A) *P. catamarcensis* (EEP-Pc) and (B) *T. fiebrigi* (EEP-Tf). The results were generated from the values of 3 independent gels. The results are expressed as mean ± SEM. ^####^*P* < 0.0001 versus Control; *P < 0.05, ***P < 0.001 and ****P < 0.0001 versus HU (H**₂**O**₂**+UV).

The propolis extracts reduced genomic DNA fragmentation at all evaluated concentrations (50–1000 μg/mL) in a concentration-dependent manner (Fig 2A-B). EEP-Pc protected genomic DNA against oxidative damage, maintaining its integrity at 87.13 ± 1.08% at a concentration of 1000 μg/mL (Fig 2A). Additionally, treatment with 1000 μg/mL of EEP-Tf maintained genomic DNA integrity at 92.82 ± 1.67% under oxidative stress conditions (Fig 2B).

### *In vivo* assays

#### Toxicity.

**Fig 3:**
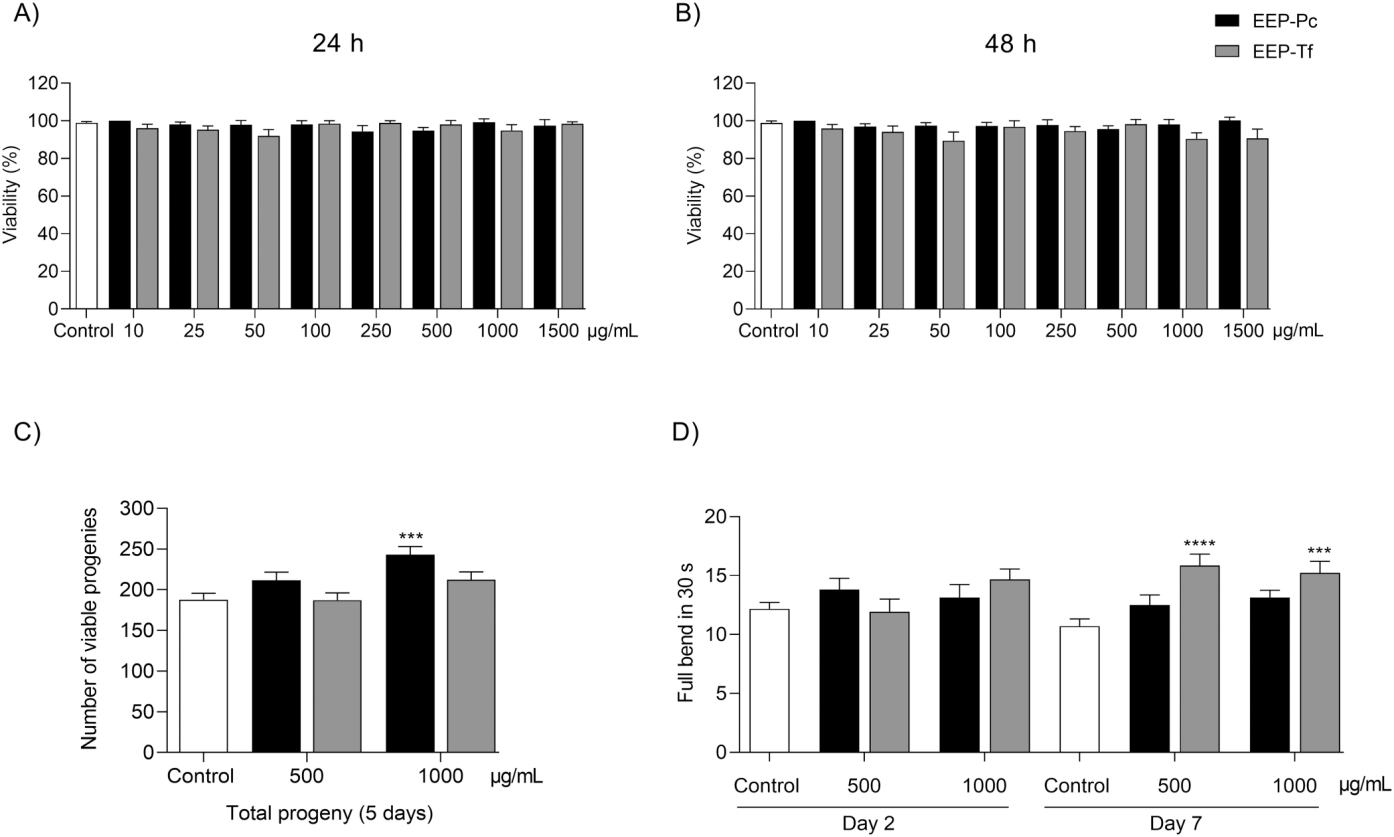
Effect of ethanolic extract of propolis from *P. catamarcensis* (EEP-Pc) and *T. fiebrigi* (EEP-Tf) in N2 *C. elegans* viability after: (A) 24 h, (B) 48 h, (C) reproduction, and (D) locomotion. Values are expressed as mean ± SEM. ***P < 0.001, ****P < 0.0001 versus Control.

The effects of EEP-Pc and EEP-Tf on *C. elegans* viability, reproduction, and mobility were considered toxicity parameters (Fig 3A-D). After 24 and 48 hours of treatment, neither extract reduced worm viability at the evaluated concentrations (10–1500 µg/mL) (Fig 3A-B). When assessing the action of the extracts on nematode reproduction, it was observed that EEP-Tf did not affect worm egg-laying. However, nematodes treated with 1000 μg/mL of EEP-Pc showed a 31.6% increase in viable progeny (Fig 3C).

Regarding the activity of the extracts on nematode mobility, it was observed that EEP-Pc did not affect the mobility of nematodes from the adult phase (day 2) to the aging phase (day 7). On the other hand, worms treated with EEP-Tf exhibited a mobility increase in the aging phase (day 7) of 37.8% and 32.3% at concentrations of 500 and 1000 µg/mL, respectively (Fig 3D).

#### Antioxidant activity.

**Fig 4:**
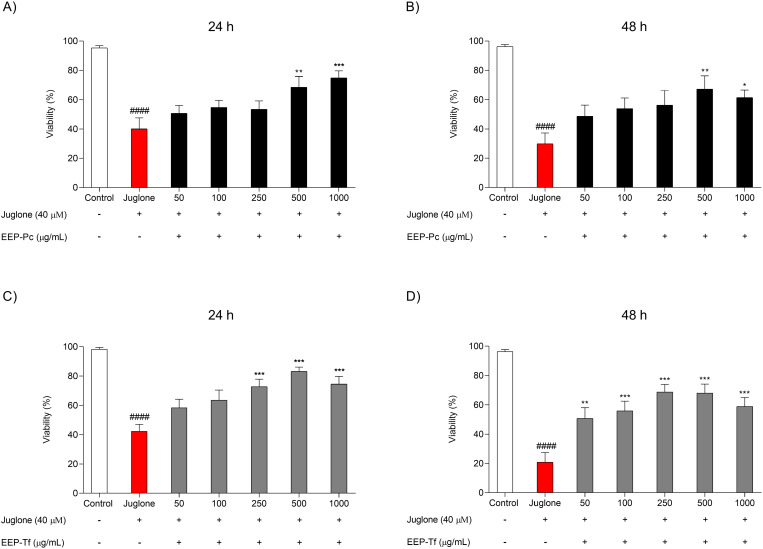
Effect of ethanolic extract of propolis from *P. catamarcensis* (EEP-Pc) and *T. fiebrigi* (EEP-Tf) against oxidative stress induced by Juglone in N2 *C. elegans:* after treatment with EEP-Pc for (A) 24 h and (B) 48 h, and after treatment with EEP-Tf for (C) 24 h and (D) 48 h. Values are expressed as mean ± SEM. ^####^*P* < 0.0001 versus Control; *P < 0.05, **P < 0.01, ***P < 0.001 versus Juglone.

The effect of EEP-Pc and EEP-Tf on Juglone-induced oxidative stress is exhibited in Fig 4A-D. EEP-Pc increased worm viability by 71% and 87% at concentrations of 500 and 1000 μg/mL, respectively, after 24 h of treatment compared to nematodes that received only Juglone (Fig 4A). After 48 h, worm viability increased by 125%, and 105% at concentrations of 500 and 1000 μg/mL, respectively (Fig 4B).

EEP-Tf increased worms’ viability by 71% and 95% at concentrations of 250 and 500 μg/mL, respectively, after 24 h of treatment (Fig 4C). After 48 h, EEP-Tf mitigated the reduction in worm viability at all evaluated concentrations. At the lowest concentration evaluated (50 μg/mL), increased viability by 143% compared to nematodes treated with Juglone only (Fig 4D).

#### Lifespan.

**Fig 5:**
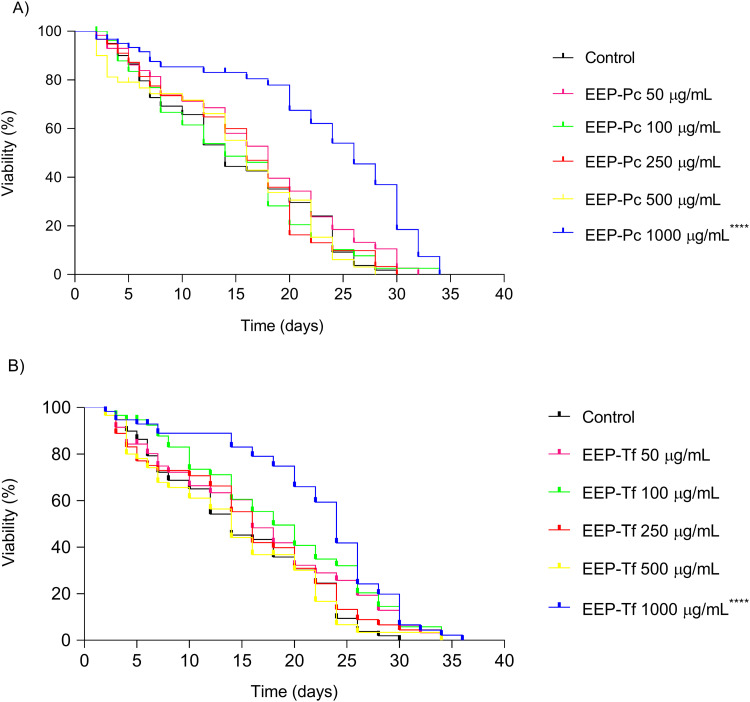
Effect of ethanolic extract of propolis on N2 *C. elegans* lifespan: (A) *P. catamarcensis* (EEP-Pc) and (B) *T. fiebrigi* (EEP-Tf). The results are expressed as mean ± SEM. ****P < 0.0001 versus Control.

The effect of EEP-Pc and EEP-Tf on the lifespan of *C. elegans* is presented in Table 3 and Fig 5A-B. Both EEP-Pc and EEP-Tf increased worm mean and maximum lifespan at the concentration of 1000 μg/mL, with EEP-Tf presenting a greater impact on nematode lifespan.

**Table 3:** Effect of ethanolic extract of propolis from *P. catamarcensis* (EEP-Pc) and *T. fiebrigi* (EEP-Tf) on *C. elegans* N2 lifespan.

Treatment (μg/mL)	Mean lifespan^a^ (days)		Maximum lifespan^a^ (days)		P value (log-rank) vs. Control^b^		Total number of nematodes	
	EEP-Pc	EEP-Tf	EEP-Pc	EEP-Tf	EEP-Pc	EEP- Tf	EEP-Pc	EEP-Tf
0	14	14	30	30	–	**–**	120	120
50	18	16	32	34	0.1826	0.1389	120	120
100	14	18	34	34	0.9769	0.0121	120	120
250	16	16	30	34	0.7606	0.4695	120	120
500	16	14	28	34	0.6811	0.7327	120	120
1000	26	24	34	36	<0.0001^*^	<0.0001^*^	120	120

* Represents statistically significant results (P<0.05) when the treated group was compared with the control group.

^a^Lifespan measured in days.

^b^The comparisons were performed by using the log-rank test (Mantel-Cox).

EEP-Pc at 100 μg/mL and 1000 μg/mL, extended the maximum lifespan by 4 days, representing an increase of 13% compared to the control (Fig 5A). EEP-Tf demonstrated a greater effect, prolonging maximum lifespan by 4 days at concentrations of 50–500 μg/mL, and by 6 days at 1000 μg/mL concentration, corresponding to a 20% increase in worm lifespan (Fig 5B). Additionally, EEP-Pc increased nematode mean lifespan by 12 days, while EEP-Tf-treated worms had their lifespan expanded by 10 days (Table 3).

#### β-amyloid peptide-induced paralysis.

**Fig 6:**
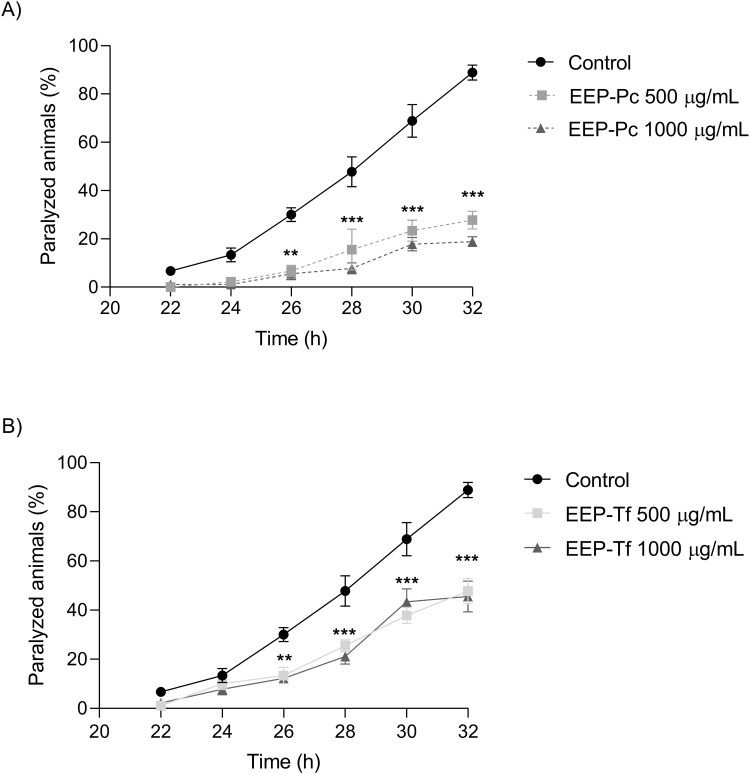
Effect of ethanolic extract of propolis onβ-amyloid-induced paralysis in CL2006 *C. elegans:* (A) *P. catamarcensis* **(EEP-Pc) and (B) ***T. fiebrigi* **(EEP-Tf).** The values are expressed as mean ± SEM. **P < 0.01; ***P < 0.001 versus Control.

When assessing the effects of the extracts on β-amyloid-induced paralysis in CL2006 mutant *C. elegans*, EEP-Pc reduced paralysis in worms by 65% and 76% at the concentrations of 500 and 1000 µg/mL, respectively, in comparison with control nematodes (Fig 6A). EEP-Tf, however, mitigated the paralysis by 39% (500 µg/mL) and 42% (1000 µg/mL), compared to control (Fig 6B).

## Discussion

Bee products, such as propolis, contain promising substances for the development of new and safe pharmacological therapies. This study is the first to show the effects of propolis from stingless bees *Plebeia catamarcensis* and *Tetragonisca fiebrigi* on oxidative stress, lifespan, and Alzheimer’s disease in the pre-clinical model *C. elegans*.

Upon evaluating the chemical constituents present in EEP-Pc and EEP-Tf, we identified that although both extracts contain phenolic compounds and terpenes, there is variation in the presence and concentration of the evaluated substances. In EEP-Pc, vanillic acid is the major phenolic compound, while in EEP-Tf, terpenes like β-amyrin, lupeol, β-amyrin and lupeol acetate, are predominant. These compounds are known for their antioxidant potential due to their chemical structure, particularly the presence of hydroxyl groups [22]. They are secondary metabolites produced naturally by plants, capable of donating electrons or hydrogen atoms to stabilize free radicals [23].

The antioxidant activity of EEP-Pc and EEP-Tf was demonstrated *in vitro* through the elimination of DPPH• free radicals, with EEP-Pc showing a lower IC_50_ compared to EEP-Tf. The better activity of this extract is probably related to the presence of vanillic acid, a phenolic acid known for its high antioxidant action [24]. Additionally, quercetin [25] and the terpenes stigmasterol [26], β-sitosterol [27] and lupeol [28]—identified in both extracts—are also known for their antioxidant properties.

Reactive oxygen species (ROS), such as superoxide anions and hydroxyl radicals, are generated by physiological processes [29], their levels are regulated by a balance of their production and neutralization. However, when this balance is disturbed, often by exogenous factors, such as toxins, environmental pollutants, and smoking, oxidative stress occurs [29]. Under these conditions, ROS can oxidize biomolecules, causing damage to lipids, proteins, and DNA, leading to physiological and morphological cellular changes, which can contribute to the development of chronic noncommunicable diseases, including neurodegenerative conditions such as Alzheimer’s disease [30–32].

In this study, EEP-Pc and EEP-Tf mitigated oxidative damage to proteins and DNA. These results may be attributed to chemical constituents in both extracts, which were capable of neutralizing reactive species *in vitro*. In the case of proteins, the compounds could protect against structure breakdown and side-chain fragmentation, potentially reducing conformational changes, structural abnormalities, or loss of protein function *in vivo* [8,33]. In DNA, phenolic acids such as vanillic acid present in EEP-Pc, and caffeic and ferulic acids present in EEP-Tf, have been described as protecting DNA against deleterious effects induced by oxidative agents [34].

In addition to *in vitro* assays used to investigate the pharmacological activities of different substances, *in vivo* experiments are widely employed to assess the efficiency and safety of natural products [35,36]. In this study, both extracts showed no negative effects on *C. elegans* viability, reproduction, and mobility, indicating their safety in this pre-clinical model.

Corroborating with the antioxidant effects observed *in vitro*, the extracts also were able to protect *C. elegans* against oxidative stress. In this model, the redox imbalance response is regulated by several pathways, and chemical substances can modulate transcriptional factors and influence the expression of different genes [37].

Among the signaling pathways, DAF-16 and SKN-1 may have been upregulated by the phenolic compounds quercetin [38], p-coumaric acid [39] and the terpene lupeol [40], identified in both extracts. In addition to these, caffeic acid [41] and luteolin [42], identified only in EEP-Tf, are also described to activate the transcriptional factor DAF-16.

The DAF-16 and SKN-1 pathways in *C. elegans*, which are respectively homologous to FOXO and Nrf2 in humans, are also related to the expression of pro-longevity factors [43]. In this study, the extracts increased the nematode lifespan. Previous studies have demonstrated that quercetin, found in both extracts, has a positive impact on the longevity of *C. elegans* [38,44]. Additionally, vanillic acid [45] and caffeic acid [41], identified only in EEP-Pc and EEP-Tf, respectively, have also been described for their pro-longevity effects in this pre-clinical model.

Furthermore, activation of DAF-16 and SKN-1 pathways is also linked with resistance to β-amyloid-induced proteotoxicity and maintenance of protein homeostasis [46,47]. Here, we assessed the protective effects of EEP-Pc and EEP-Tf in CL2006 nematodes, which are β-amyloid-aggregation mutants that exhibit the paralysis phenotype. In these worms, the β-amyloid plaque accumulation may be verified by the loss or reduced motor function [14]. The extracts reduced β-amyloid-induced paralysis on CL2006 *C. elegans*, with emphasis on the protection promoted by EEP-Pc, which showed greater paralysis reduction. This result may be attributed to vanillic acid, which has been described as attenuating the paralysis phenotype associated with β-amyloid aggregation in *C. elegans*, suggesting that this compound improves protein homeostasis [45]. Meanwhile, the attenuation promoted by EEP-Tf treatment in paralysis may be related to the presence of caffeic acid, which was previously described as an agent capable of reducing β-amyloid toxicity in *C. elegans*, via activation of DAF-16 and its subsequent targets SOD-3 and GST-4 [41]. Additionally, quercetin already demonstrated efficient anti-amyloid *in vitro* activity [38].

β-amyloid aggregation is a known precursor of Alzheimer’s disease [1]. In a previous study, Sabogal-Guáqueta et al. [48] assessed the quercetin effect on behavior in triple transgenic mouse models of Alzheimer’s disease, and the authors were able to verify a reduction in β-amyloid accumulation along with improved behavioral performance.

The correlation between aging and Alzheimer’s disease indicates the presence of overlapping molecular and cellular processes that can be targeted by substances aimed at preventing and/or treating neurodegenerative conditions. Our data suggests that *P. catamarcensis* and *T. fiebrigi* propolis have promising applications in pharmacological interventions for the prevention and treatment of oxidative stress-associated diseases, especially Alzheimer’s disease.

## Supporting information

S1 Fig Base peak chromatogram of HPLC of ethanolic extract of propolis from P. catamarcensis (EEP-Pc) and T. fiebrigi (EEP-Tf): (1) vanillic acid, (2) caffeic acid, (3) p-coumaric acid, (4) ferulic acid, (5) quercetin, (6) luteolin and (7) apigenin. (DOCX)
