# Supplementary material for: Plebeia catamarcensis and Tetragonisca fiebrigi (Hymenoptera, Apidae) propolis promotes longevity and anti-Alzheimer effects in Caenorhabditis elegans
Source: PLoS One. 2025 Jun 3;20(6):e0321487. doi: 10.1371/journal.pone.0321487 (PMC12133018; doi:10.1371/journal.pone.0321487)
Supplement: S1 Fig — (DOCX) [file pone.0321487.s001.docx]

**S1 Figure**


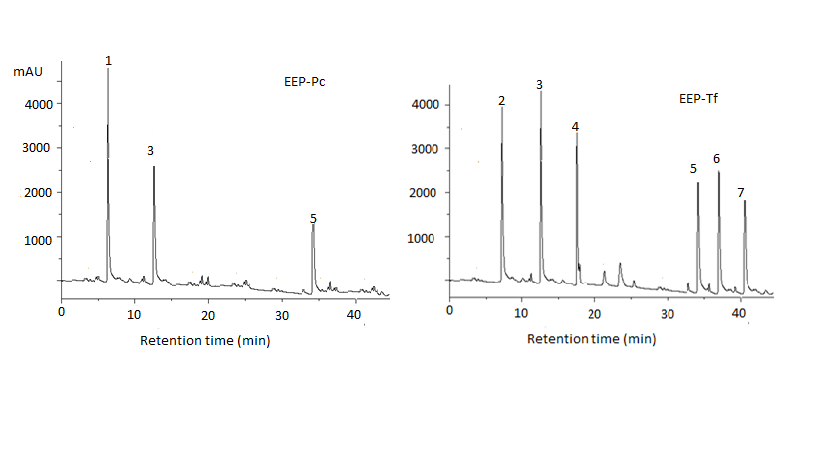


**S1 Fig. Base peak chromatogram of HPLC of ethanolic extract of propolis from *P. catamarcensis* (EEP-Pc) and *T. fiebrigi* (EEP-Tf):** (1) vanillic acid, (2) caffeic acid, (3) p-coumaric acid, (4) ferulic acid, (5) quercetin, (6) luteolin and (7) apigenin.
